# If AI writes the paper, who validates the plankton?: a call to reinvest in empirical expertise before the validation gap becomes irreversible

**DOI:** 10.1093/plankt/fbag045

**Published:** 2026-06-30

**Authors:** Albert Calbet

**Affiliations:** Institut de Ciències del Mar, CSIC, Department of Marine Biology and Oceanography, Pg. Marítim de la Barceloneta 37–49, 08003 Barcelona, Spain

**Keywords:** artificial intelligence, automated plankton imaging, empirical validation, experimental biology, natural history, plankton taxonomy

## Abstract

Artificial intelligence is already reshaping the production of scientific text, literature synthesis, coding and image-based biological analysis. In plankton research, where automated imaging, molecular surveys and large ecological datasets are expanding rapidly, the critical bottleneck will increasingly be empirical validation: knowing organisms, recognizing artefacts, maintaining cultures, designing biologically meaningful experiments and judging whether automated outputs remain faithful to living plankton. Yet these are precisely the skills that many evaluation systems have allowed to erode. Taxonomy, fieldwork, experimental manipulation, natural history and specimen curation have lost institutional status in a culture that rewards speed, scale and publication volume. The rise of AI makes this loss urgent rather than merely unfortunate. This Horizons article argues that AI will not eliminate the need for plankton experts; it will expose how dangerous it is to lose them. Journals, funders and training programs should therefore revalue empirical expertise, not out of nostalgia, but because taxonomic, experimental and organismal judgment will soon become the bottleneck on which the credibility of AI-assisted plankton science depends.

## INTRODUCTION

I have spent my career doing kinds of biology that are difficult to reduce to metrics: identifying planktonic organisms, maintaining cultures, running controlled experiments on living animals, and knowing from experience when a culture is healthy, stressed or already artefactual. These skills are slow to develop. They are difficult to count. They do not map neatly onto publication numbers, citation metrics or automated productivity indicators. Yet they are often the difference between a biologically meaningful result and a polished but unreliable paper.

Artificial intelligence (AI) is now reshaping the production of scientific text. Large language models can already assist with literature searches, synthesis, statistical coding and manuscript drafting, and their use in research workflows will continue to expand (van Dis *et al.,* 2023; Messeri and Crockett, 2024). In plankton ecology, AI is also accelerating image classification, anomaly detection and the processing of large observational datasets. Most commentary frames this transformation as a threat to scientists. I want to argue something different: AI is not a new problem for empirical biology. It is a mirror that makes an existing problem impossible to ignore.

If writing, analysis and computational synthesis become easier and cheaper (Table I), then what we have allowed to erode becomes the bottleneck instead: organism handling, taxonomic judgment, experimental design, field observation and the ability to recognize when an apparently elegant output is biologically wrong. That realization should generate urgency. This article is a call to action for plankton research and for the broader biological community, because plankton ecology sits at the intersection of taxonomy, experiments, automated observation, molecular surveys and global modelling. It is therefore an ideal field in which to ask a simple question: if AI writes the paper, who validates the biology behind it?

**Table I:** Tasks increasingly assisted by AI compared with empirical contributions that remain essential in plankton research and experimental biology

Tasks increasingly assisted by AI	Empirical contributions requiring expert validation
Literature search and synthesis	Organism identification and taxonomic validation
Statistical analysis and coding	Experimental design, execution and troubleshooting
Manuscript drafting and editing	Field observation and ecological interpretation
Figure preparation and formatting	Recognition of artefacts and methodological failure
Reference organization	Judgement about biological plausibility
Hypothesis generation from existing data	Validation of model outputs against living systems
Automated image classification	Specimen collection, curation and voucher linking

The table is not intended to suggest that AI-assisted tasks require no expertise, but to indicate where the validation bottleneck will increasingly lie as AI capabilities expand

## WHAT CHANGES WHEN WRITING BECOMES CHEAP?

Scientific papers are compressed representations of empirical work. Yet much of the labour involved in producing a paper is often spent on tasks surrounding the empirical core: framing arguments, restructuring introductions, adapting to reviewer conventions, writing code, producing figures and reformatting references. AI systems are particularly well suited to accelerate these text-centred and pattern-centred tasks.

The risks of AI-assisted writing are real and well documented: fabricated citations, overgeneralized conclusions and hallucinated findings (Walters and Wilder, 2023). But these weaknesses clarify rather than contradict the broader point. As the cost of producing manuscripts falls, the manuscript itself becomes a less reliable signal of scientific quality on its own. What remains irreducible is the empirical work behind the text: the species identity, the experimental design and implementation, the data provenance and the biological plausibility of the conclusion.

If AI writes part of the paper, the scientist’s central responsibility shifts toward a harder question: does this paper deserve to exist? Was the organism correctly identified? Was the experiment soundly designed and conducted? Was the conclusion actually supported by the data, or does it extend beyond what the system showed? These questions cannot be answered reliably by a language model alone. They require judgment formed through direct contact with living systems.

## THE VALIDATION BOTTLENECK IN PLANKTON RESEARCH

Plankton research shows especially clearly why empirical validation matters. Automated imaging systems such as FlowCam, ZooScan, Imaging FlowCytobot and underwater vision profilers have transformed the scale at which plankton communities can be observed. Machine-learning tools can process image collections far faster than a human analyst and can help detect patterns that would otherwise remain hidden (Lombard *et al.,* 2019; Irisson *et al.,* 2022; Eerola *et al.,* 2024). These advances are not peripheral to the future of the field; they are becoming part of its observational infrastructure.

**Fig. 1:**
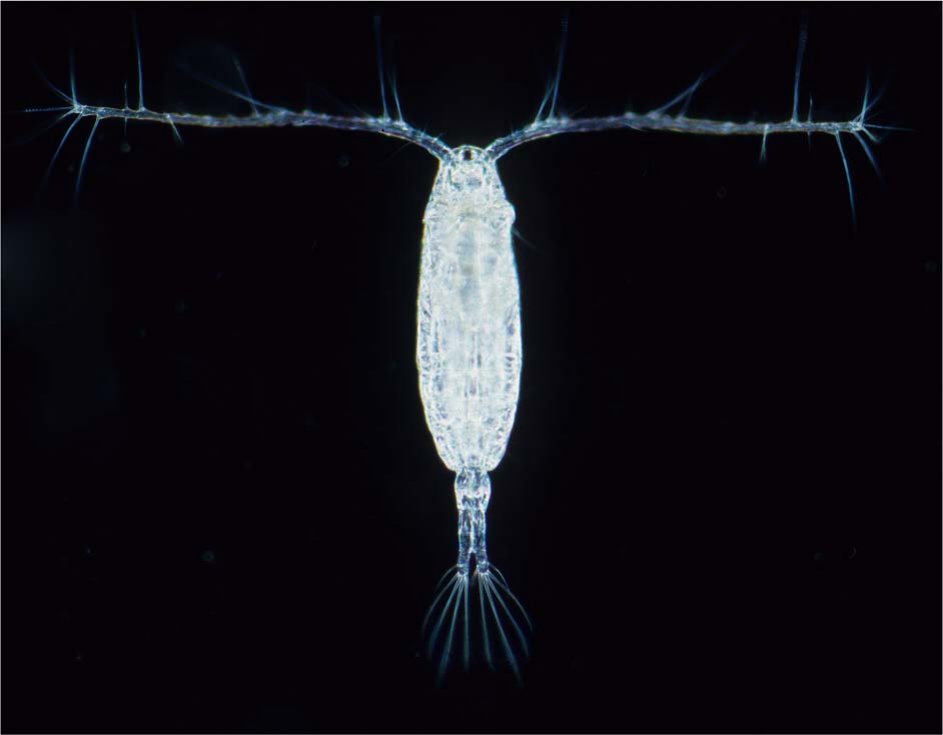
*Acartia margalefi* was long classified as *Acartia clausi* until it was formally described by Alcaraz in 1976 (Alcaraz, 1976). Distinguishing between the two species required careful examination of fine morphological traits, including details of the fifth swimming leg. Current machine-learning systems can assist taxonomic workflows, but they cannot replace expert judgement unless trained, audited and corrected against reliable human-validated reference material.

Yet automated classification does not remove the need for plankton expertise (Fig. 1). It changes where that expertise is needed. Plankton image datasets are taxonomically heterogeneous, instrument-dependent, often imbalanced among classes, and frequently filled with rare organisms, damaged specimens, detritus, aggregates, artefacts and ambiguous particles. A classifier trained on uncertain categories can reproduce and amplify uncertainty. A model that sorts particles efficiently can still confuse an ecologically meaningful taxon with a similar-looking fragment. A global synthesis built from automated labels can only be as trustworthy as the taxonomic and methodological validation behind those labels.

The same logic applies to molecular approaches. Environmental DNA, metabarcoding and comparative genomic datasets have expanded the scale of plankton biodiversity research, but their biological meaning depends on the quality of reference databases, voucher linkage and taxonomic interpretation. Public sequence databases and barcode libraries may contain taxonomic errors or poorly documented entries (Harris, 2003; Collins and Cruickshank, 2013), and genome assemblies may lack linked voucher specimens that allow independent verification of what was sequenced (Buckner *et al.,* 2021). Molecular tools do not replace taxonomy and natural history; they amplify the consequences of losing them.

This is why the coming bottleneck is not only computational. It is empirical. AI can help classify, summarize, model and suggest. It cannot, by itself, ensure that the organism was the organism we thought it was, that the culture was physiologically normal, that the experimental design made biological sense, or that the ecological interpretation remained faithful to the living system rather than to the structure of the data used to train the model.

## THE SKILLS WE HAVE BEEN DEVALUING

Biology has been moving away from organisms for decades. Taxonomy, natural history, fieldwork and hands-on experimental biology have progressively lost institutional status in a scientific culture that rewards speed, scale, novelty and publication volume (Lindenmayer and Likens, 2011; Tewksbury *et al.,* 2014). The taxonomic impediment—the shortage not of tools, but of trained taxonomists—has been described for more than 20 years and has not been resolved (Wheeler *et al.,* 2004; Engel *et al.,* 2021). Fieldwork-based investigations in conservation science have declined substantially since the 1980s (Ríos-Saldaña *et al.,* 2018). The “extinction of experience among ecologists”, as Soga and Gaston (2025) recently named it, is now measurable and documented.

Experimental biology faces the same problem. Experimental skill is partly tacit: knowing when a copepod culture is stressed, when a grazer is feeding normally, when a bottle incubation has become unrealistic, when a preparation is behaving abnormally, or when a statistically significant response is biologically trivial. This knowledge rarely appears fully in methods sections. It lives in the hands and judgment of practitioners, and it is transmitted through direct experience. It is lost when training programs reduce laboratory contact, shorten fieldwork placements and treat organismal biology as a foundation course rather than a lifelong competence.

These are not peripheral skills. They are the foundation on which computational biology stands. A machine-learning classifier does not solve the identification problem if its training data are mislabeled. An environmental DNA pipeline does not remove the need for taxonomy if the reference database is incomplete or poorly linked to voucher specimens. A species distribution model is not reliable if occurrence records contain misidentifications. Bortolus (2008) described how taxonomic errors can generate error cascades through ecological interpretation and management, and Costa *et al.* (2015) showed that misidentified species can distort species distribution model outputs. The more elaborate the computational superstructure, the harder such errors may be to detect.

## TESTABLE PREDICTIONS AND RESEARCH DIRECTIONS

The argument advanced here leads to testable predictions for plankton research. First, as automated plankton imaging and AI-assisted classification expand, the limiting factor will shift from image acquisition to expert-validated reference material. Datasets with stronger taxonomic curation, expert audit trails and transparent uncertainty estimates should produce more stable ecological interpretations than datasets lacking such validation.

Second, taxonomic and methodological errors should propagate nonlinearly through plankton ecology. A modest rate of misclassification may have limited consequences for broad biomass estimates but large effects on trait-based inference, trophic interpretation, bloom detection, biogeographic comparisons or modelled carbon fluxes if the errors involve ecologically distinct taxa. This prediction can be tested through sensitivity analyses that introduce realistic rates of taxonomic error into community datasets, food-web analyses and biogeochemical models.

Third, AI-generated hypotheses and sampling designs will be most useful when they are audited by researchers with field, taxonomic and experimental experience. A statistically optimized sampling design may still be impractical at sea, insensitive to organismal behaviour, blind to preservation artefacts or irrelevant to the ecological process of interest. Controlled reclassification exercises, blind expert audits of automated datasets, comparisons between voucher-linked and non-voucher-linked molecular records, and experiments designed jointly by computational and organismal specialists would allow these predictions to be evaluated rather than merely asserted.

## WHY PLANKTON RESEARCHERS ARE ESPECIALLY WELL PLACED TO ACT

Plankton researchers work daily with the tension between scale and detail. The field needs satellites, imaging systems, omics, automated classifiers and global models, but it also needs people who can recognize a damaged tintinnid lorica, distinguish similar copepod species, maintain fragile cultures, understand bottle effects, judge whether a grazing response is plausible and know when an apparently clean dataset conceals a biological artefact. Few areas of biology make the dependence of large-scale inference on small-scale expertise so visible.

This dependence will only grow. In the short term, AI will mainly accelerate tasks that are already semi-automated: sorting plankton images, flagging likely taxa, detecting anomalies and helping experts prioritize samples that need inspection. In the medium term, AI systems will increasingly combine images, molecular data, environmental metadata and historical observations to suggest community shifts, functional traits, sampling priorities and candidate experiments. Recent benchmark work already shows that machine learning can substantially accelerate plankton image processing, but also that performance depends strongly on training data, class structure and the biological meaning of the categories being predicted (Panaïotis *et al.,* 2026).

In the longer term, AI may propose experiments and sampling designs that are technically sophisticated and statistically optimized. This does not remove the need for empirical expertise; it changes where it is needed. The critical human role will increasingly be to define which biological questions matter, to recognize when an apparently optimal design is experimentally unrealistic or ecologically trivial, and to validate whether AI-generated classifications, hypotheses and interpretations remain faithful to living plankton.

## A CALL TO ACTION

The trajectory described above is not inevitable. But changing it requires deliberate decisions by journals, funders and training programs.

Journals should lead by revaluing empirical contributions. Taxonomic revisions, methods papers, replication studies, negative results, expert-validated image libraries and voucher-linked datasets are essential to the scientific record, but many evaluation systems treat them as second-tier outputs. Researchers, especially early-career researchers under publication pressure, are rationally incentivized to avoid them. Journals could change this signal by commissioning, publishing and visibly celebrating such work. An article that corrects a widely used misidentification is a service to the field, not a consolation prize.

Funders should treat empirical infrastructure as what it is: infrastructure. Natural history collections, long-term field stations, culture facilities, monitoring programs and long-term experiments are not ordinary projects to be funded briefly and then discontinued. Their value compounds over time in ways that short grant cycles cannot accommodate. Forbes *et al.* (2025) recently reported substantial declines in global specimen collection rates over recent decades—precisely as new analytical tools increase the value of those specimens. Funders should create dedicated, stable mechanisms to sustain empirical infrastructure and should require empirical validation components in computational and AI-assisted grant proposals.

Training programs should protect taxonomy, fieldwork and experimental design as required graduate competencies, not optional specializations. The student who learns to handle and identify organisms during a PhD will be better equipped to evaluate computational claims about those organisms for the rest of their career. Rafiq *et al.* (2024) argue that long-term fieldwork is hindered by institutional barriers, including publish-or-perish culture and funding limitations, and propose broader evaluation metrics and stronger support for early-career researchers. These reforms are relevant to plankton ecology and experimental biology equally. PhD timelines, supervision models and assessment criteria should be revised to accommodate the pace of rigorous empirical research.

## CONCLUSION

I am not arguing against AI. I used AI tools during the preparation of this manuscript to help review grammar and style, and I expect such tools to become increasingly useful in scientific work. The argument is instead against what will happen to plankton research, and to biology more broadly, if we do not act while we still can. AI is not the cause of the erosion of empirical expertise; it is a clarifying force that makes the cost of that erosion visible.

Biological sciences faces a fork in the road. One path leads to a science that is technically impressive but increasingly detached from organisms: more papers, more models and more databases, but fewer scientists who can say whether the biology is right. The other leads to something more durable: a science in which AI amplifies empirical expertise rather than substituting for it, and in which the scientist who knows the organism is recognized as indispensable.

Most of the scientific community already believes the second path is worth taking. The question is whether we are willing to say so loudly enough, and to change the systems that make it difficult to walk. If we are, the rise of AI could be one of the best things that has happened to plankton research in a generation—not because it replaces what we do, but because it finally makes visible just how irreplaceable empirical expertise is.

## Data Availability

No data were produced in this manuscript.
